# TRANSCRANIAL DIRECT CURRENT STIMULATION MODULATES BRAIN ACTIVITY DURING DUAL-TASK STANDING IN OLDER ADULTS

**DOI:** 10.1093/geroni/igad104.0552

**Published:** 2023-12-21

**Authors:** Melike Kahya, Natalia Gouskova, On-Yee Lo, Junhong Zhou, Davide Cappon, Alvaro Pascual-Leone, Lewis Lipsitz, Brad Manor

**Affiliations:** Marcus Institute for Aging Research, Boston, Massachusetts, United States; Hebrew SeniorLife, Roslindale, Massachusetts, United States; Hebrew SeniorLife/ Harvard Medical School, Boston, Massachusetts, United States; Harvard Medical School/Hebrew SeniorLife, Roslindale, Massachusetts, United States; Marcus Institute for Aging Research, Boston, Massachusetts, United States; Hebrew SeniorLife, Roslindale, Massachusetts, United States; Hebrew SeniorLife, Boston, Massachusetts, United States; Hebrew SeniorLife/Harvard Medical School, Amherst, Massachusetts, United States

## Abstract

In older adults, performing an attention-demanding cognitive task while standing comes with a ‘cost’ (i.e., decrement) to postural control. Transcranial direct current stimulation (tDCS) designed to facilitate the excitability of the left dorsal-lateral prefrontal cortex (L-DLPFC) and motor cortex (M1) mitigates this dual-task cost (DTC). The effects of tDCS on underlying brain activity measured by electroencephalography (EEG) during dual-tasking, however, is unknown. We hypothesized that tDCS would decrease the DTC of EEG-measured alpha and gamma power, as these frequencies have been linked to attentional processing and their attenuation is believed to reflect more efficient and/or effective postural control. Thirty participants received 20-minutes of tDCS targeting the L-DLPFC and M1, or sham (i.e., control) stimulation, in random order, on two separate visits. Before and immediately after each stimulation, EEG and postural sway area were recorded as participants completed trials of standing with and without verbalized serial subtractions. EEG alpha and gamma power were derived from channels corresponding to the L-DLPFC and M1 areas. The DTC (absolute change from single to dual-tasking) for both EEG and sway outcomes were calculated. tDCS, compared to sham, decreased the DTC to gamma power (p=0.04) and sway area (p=0.04). No pre-to-post stimulation changes were observed for alpha power (p=0.42). Participants who demonstrated a greater decrease in the DTC to gamma power after tDCS exhibited a greater reduction (improvement) in the DTC to sway area (r=0.66, p=0.002). The results suggest that the tDCS-induced changes in gamma-frequency activity were associated with improved dual-task standing balance in older adults.

